# Role of coefficient of thermal expansion on bond strength of ceramic veneered yttrium-stabilized zirconia

**DOI:** 10.4317/jced.54605

**Published:** 2018-03-01

**Authors:** Niwut Juntavee, Chollada Dangsuwan

**Affiliations:** 1 Department of Prosthodontics, Faculty of Dentistry, Khon Kaen University, Khon Kaen, Thailand; 2 Division of Biomaterials and Prosthodontics, Faculty of Dentistry, Khon Kaen University, Khon Kaen, Thailand

## Abstract

**Background:**

Incompatible coefficient of thermal expansion (CTE) is supposed to be a reason for chipping of ceramic veneered zirconia. This study evaluates the effect of veneering ceramic at varied CTE on bond strength to zirconia.

**Material and Methods:**

Zirconia disks (Z, Ø 10 mm, 1.0 mm thickness) were prepared from Y-TZP (Cercon®) and sintered at 1350°C for 6 hours. All zirconia disks were veneered with ceramics ((Ø 7.0 mm, 1.5 mm thickness) with varied CTE including VITADur® alpha (VDα), VITAVM®7 (VM7), VITAVM®9 (VM9), Cercon® ceramkiss (CCK), IPSe.max® ceram (IeC), and IPS dSIGN® (IdS) (n=15). The specimens were thermo-cycled (5-55 °C, 500 cycles) prior to determine the shear bond strength on a universal testing machine. The veneering ceramic and zirconia rods (Ø 4 mm, 30 mm length) were prepared for CTE evaluation. ANOVA and Tukey’s multiple comparisons were used to determine the statistically significant difference (α=0.05). Weibull analysis was applied for survival probability, Weibull modulus (m), and characteristics strength (σo) of the shear bond. The interfaces were microscopically examined. The phase transformation of zirconia was determined using X ray diffraction.

**Results:**

The mean±sd (MPa), m, and σo of bond strength were 20.45±2.32, 9.25, and 21.53 for Z-VDα, 19.47±4.53, 4.66, and 20.31 for Z-VM7, 21.05±3.96, 5.61, and 21.88 for Z-IeC, 25.85±2.74, 9.93, and 27.15 for Z-VM9, 25.82±4.39, 6.27, and 27.06 for Z-CCK, and 2.96±0.73, 4.11, and 3.28 for Z-IdS. The CTE (×10-6/°C) were 10.80, 7.83, 7.87, 9.86, 9.93, 10.03, and 12.95 for Z, VDα, VM7, IeC, VM9, CCK, and IdS. The bond strength was significantly affected by the CTE difference (*p*<0.05). The t→m phase transformation related with the CTE difference.

**Conclusions:**

The CTE’s differences induced stress that affected the bond strength. CTE’s compatibility of veneering ceramic to zirconia is crucial for enhancing the bond strength. The CTE difference approximately 0.77-0.87×10-6/°C was recommended.

** Key words:**Bond strength, coefficient of thermal expansion, zirconia.

## Introduction

The celebrity of using all ceramic restoration has been increasing as they provide highly desirable esthetic appearance and extreme biological compatibility. Patients often request for metal-free restoration, which makes ceramic the most preferred restoration choice for reconstruction. Several new dental ceramics have been developed with improved strengths to withstand the stress from the physiologic masticatory function, which are being used as long-span fixed prostheses (1). Among contemporary ceramic materials, yttria-stabilized tetragonal zirconia polycrystalline (Y-TZP) has recently been introduced as an alternative to metal substructure, as it possesses aesthetic properties, biological compatibility, less plaque accumulation, and minimal thermal conductivity in addition to the superior flexural strength, and fracture toughness (2,3). A unique characteristic of transformation-toughening phenomenon of Y-TZP has been reported to be its efficient ability to inhibit crack propagation (4,5). The zirconia restorations are fabricated via the process of computer-aided design and computer-aided manufacturing (CAD-CAM) technology upon milling partially sintered zirconia blank, and further sintered to achieve fully strong restorations. However, Y-TZP is relatively opaque and needs a proper veneering ceramic to achieve a reliable natural-looking restoration (6,7). Failures of ceramic veneered zirconia were reported with high incidence of fracture, chipping, and delamination of veneering ceramic from zirconia substructure (8-11). The potential failure causes are associated with an improper substructure design, the presence of critical flaws, and thermal-related interfacial residual stresses (12-15). The residual stress generated is a result of the difference in coefficient of thermal expansion (CTE) between the veneering ceramic and zirconia, which likely induces zirconia phase transformation that affects the bond strength (16-19). The low thermal conductivity of zirconia also engenders thermal accumulation upon cooling process of the ceramic firing and results in a high tensile stress at the interface (20-22).

The bond firmness between zirconia and ceramic that is partly related with the compressive force generated is because of appropriate difference of CTE’s value of veneering ceramic and zirconia (23-26). The bond strength was compromised by excessive residual stresses generating from a CTE mismatch (17,27,28). The optimal difference of CTE’s value between zirconia and veneering ceramic becomes a practical interest in promoting bond strength, which has not been reported. This study aims at evaluating the effect of veneering ceramics that possessed different CTE regarding shear bond strength to zirconia. It was hypothesized that veneering ceramics possessed varied CTE, which significantly influenced the shear bond strength of the zirconia substructure.

## Material and Methods

The shear bond strengths of six ceramics, based on different CTE, including VITADur® alpha (VDα, Vita Zahnfabrik, Bad Sackingen, Germany), VITAVM®7 (VM7, Vita Zahnfabrik, Bad Sackingen, Germany), VITAVM®9 (VM9, Vita Zahnfabrik, Bad Sackingen, Germany), Cercon®ceramkiss (CCK, Degudent GmbH, Hanau-Wolfgang, Germany), IPSe.max® ceram (IeC, Ivoclar Vivadent, Schaan, Liechtenstein), and IPS dSIGN® (IdS, Ivoclar Vivadent, Schaan, Liechtenstein) veneered to Cercon® zirconia (Z, Degudent GmbH, Hanau-Wolfgang, Germany) were evaluated.

-Zirconia Specimen Preparation

Ninety zirconia disks (12.0 mm in diameter and 1.2 mm in thickness) were prepared into disk shape from partially sintered Y-TZP blank using precision machine (Isomet® 1000, Buehler, IL, USA), and sintered in the furnace (inFire® HTC speed, Sirona, Bensheim, Germany) at the recommended temperature of 1350ºC for 6 hours, to derive fully sintered zirconia disks (10.0 mm in diameter and 1.0 mm in thickness) due to 20% volumetric sintering shrinkage.

-Ceramic Veneering Technique

All zirconia disks were randomly divided into six groups (15 disks per group) to be veneered with VDα, VM7, VM9, CCK, IeC, and IdS ceramic ( Table 1). The zirconia disks were coated with a thin layer of opaque ceramic and fired in a furnace (Programmat® P100, Ivoclar Vivadent, Schaan, Leichtenstein). The creamy mixed consistency of dentine ceramic was applied onto the zirconia disks, condensed with ultrasonic condenser (3M Unitek, St. Paul, MN, USA), and fired in a furnace in accordance with the manufacturer’s instruction. The ceramic veneering technique was allowed for firing no more than three times to derive final dimension (7.0 mm in diameter and 1.5 mm in thickness), which was then glazed.

**Table 1:**
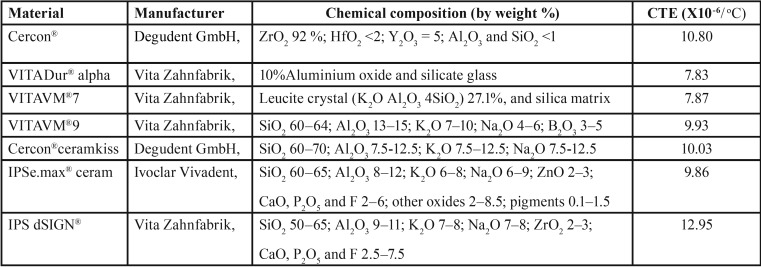
Chemical composition, coefficient of thermal expansion (CTE) of zirconia and veneering ceramics.

-Thermal Cycle Technique

The specimens were subjected to thermo-cycle process for 500 cycles between 5°C and 55°C for 30 seconds of immersion in each temperature bath, and 5 seconds for specimen transfer prior to the assessment of the shear bond strength.

-Evaluation for Shear Bond Strength

Each specimen was evaluated for its shear bond strength by subjecting it to a compression shear apparatus on a universal testing machine (Lloyd®LR 30k, Lloyd, Leicester, England). The shear load was applied at the zirconia-ceramic interface at crosshead speed of 0.5 mm/min. The loads at failure were recorded and calculated for shear bond strength (σ, MPa) by dividing the failure load (P, newton) by the area of interface (A, mm2), as shown in Equation 1: σ=P/A ……………….Equation 1.

-Determination of Coefficient of Thermal Expansion

The veneering ceramics and zirconia were prepared into a round rod shaped specimens (4 mm in diameter, 30 mm in length). The creamy mixed consistency of veneering ceramic was placed, condensed into a silicone mold, and then removed for firing and glazing process. The zirconia rod was prepared from partially sintered Y-TZP blank and sintered in the furnace to derive the final dimension. The horizontal single rod dilatometer (DIL 402 PC, NETZSCH-Gerätebau GmbH, Selb, Germany) was used to determine the heating and cooling curve for each specimen from 25°C to 500°C at the rate of 5°C per minute. The linear CTE was determined by using Proteus® software version 4.7 (NETZSCH-Gerätebau GmbH, Selb, Germany).

Microscopic Examination

The de-bonding surfaces were examined under optical stereomicroscope (Nikon, Melville, NY, USA) and scanning electron microscope (SEM, S-3000N, Hitachi, Tokyo, Japan) for characterizing the bonding failure’s mode. The zirconia-ceramic interface for each group was examined with SEM, and energy dispersive x-ray spectroscopy (Oxford instrument, Oxfordshire, United Kingdom).

-Evaluation of Crystalline Structure

The crystalline phases of ceramic veneered zirconia was determined for the relative amount of monoclinic (m) and tetragonal (t) phase of Y-TZP using the X-ray diffraction (PW 1830, Philips, Almalo, Netherland). The specimens were scanned with copper k-alpha (Cu Kα) radiation from the 2θ degree of 20–40o with 0.02o step size at every 2 seconds’ interval. The phase was analyzed in comparison to the known standard database of the joint committee on powder diffraction standards (JCPDS), and calculated for corresponding d-values using Bragg, as shown in Equation 2 (29): λ = d2 sin θ …………..Equation 2.

Where: λ is the X-ray wavelength (0.15418 nm for CuKa), d is normal distance of planes with the Miller indices (hkl), and θ is the Bragg angle.

**Figure 1:**
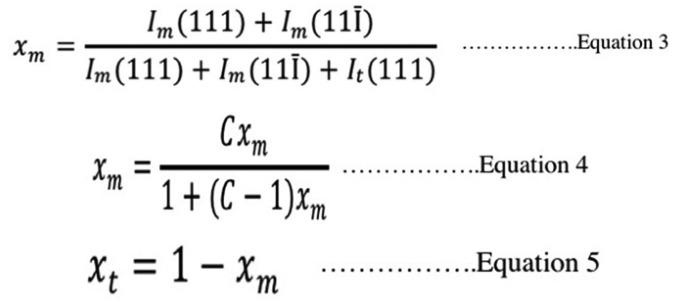
Equation 3,4,5.

The ratio of m- to t- phase was determined by the peaks’ intensities using X’Pert Plus software (Philips, Almelo, Netherland). The mass fraction of m-phase to the total zirconia phase content was calculated from Garvie-Nicholson formula, as shown in Equation 3, and further corrected for nonlinearity using Toraya formula, as shown in Equations 4 and 5 (30), (Fig. 1).

Where: Im and It: integral intensities of monoclinic and tetragonal phase

C: composition-dependent correction factor (C = 1.32)

Xt and Xm: the Toraya-corrected mass fraction of tetragonal and monolithic zirconia

-Statistical Analysis of Data

The data was statistically analyzed using SPSS/PC Version 20 software (IBM, Armonk, NY, USA). An analysis of variance (ANOVA) was used to determine the significant differences of shear bond strength upon different ceramics. Post-hoc Tukey HSD multiple comparison was used to determine the difference between groups at 95% level of confidence. Weibull analysis was performed to determine the bond strength’s reliability using Weibull++®statistics (ReliaSoft, Tucson, AZ, USA), and estimated the Weibull modulus (m) from Equation 6 and from a slope of the straight line plotted between ln{ln(1/Ps(Vo)} against m ln(σ/σo). Ps (VΟ)=exp{-(σ⁄σΟ)m } …………..Equation 6

Where: Ps (Vo) is the probability of survival as the fraction of identical sample; Vo is the volume of the sample;σ is the shear strength; σo is the Weibull characteristic strength; and m is Weibull modulus.

## Results

**Figure 2:**
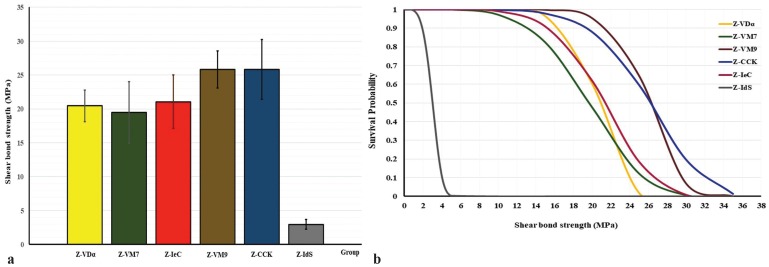
A) Bar chart representing the comparison of shear bond strength, and B) line chart representing the comparison of Weibull survival probability of shear bond strength for Cercon® zirconia (Z) veneered with Cercon® ceramkiss (CCK), VITADur® alpha (VDα), VITAVM®7 (VM7), IPSe.max® ceram (IeC), VITAVM®9 (VM9), and IPS dSIGN® (IdS).

**Figure 3:**
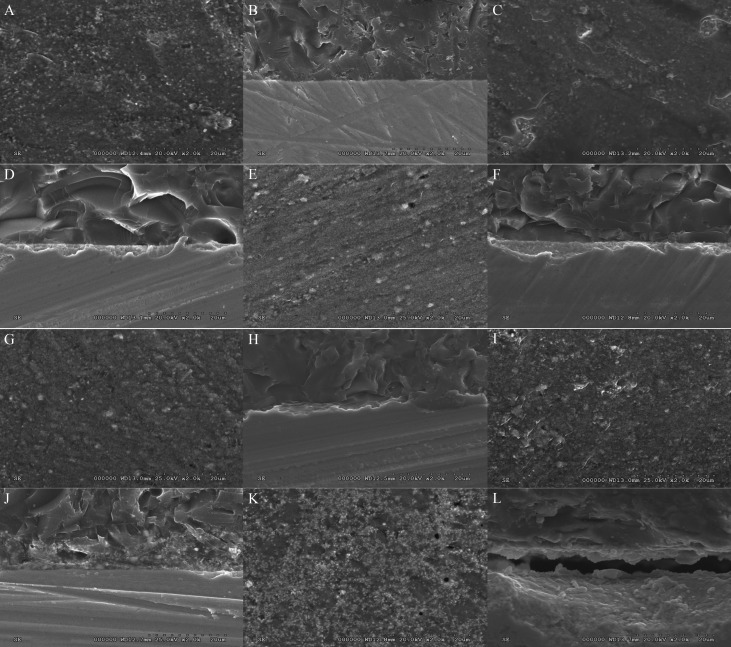
SEM of zirconia (Z) surface after de-bonded from VDα (A), VM7 (C), IeC (E), VM9 (H), CCK (I), and IdS (K) veneering ceramic, and SEM of zirconia-ceramic interface for Z- VDα (B), Z-VM7 (D), Z-IeC (F), Z-VM9 (H), Z-CCK (J), and Z-IdS (L) at X2000 magnification.

The mean, standard deviation, 95% confidence interval, Weibull’s modulus, and characteristic strength for shear bond strength for each group is presented in  Table 2 and Figure 2(A). The highest bond strength was demonstrated in the group Z-VM9 (25.85±2.74 MPa), followed by Z-CCK (25.82±4.39 MPa), Z-IeC (21.05±3.96 MPa), Z-VDα (20.45±2.32 MPa), Z-VM7 (19.47±4.53 MPa), and Z-IdS (2.96±0.73 MPa). The evaluated results of the CTE (×10/°C) for Z, VDα, VM7, IeC, VM9, CCK, and IdS were 10.80, 7.83, 7.87, 9.86, 9.93, 10.03, and 12.95, respectively, as presented in Table 1. An analysis of variance (ANOVA) indicated a statistically significant difference of shear bond strength because of varied CTE of the veneering ceramics (*p*<0.05) ( Table 3). Post-hoc Tukey multiple comparisons indicated significant difference in the shear bond strength among the groups of ceramic veneered zirconia (*p*<0.05), except for the groups of Z-VM7, Z-VDα, and Z-IeC (*p*>0.05) ( Table 4). Weibull analysis indicated the shear bond’s characteristic strength ranking from the highest to lowest as Z-VM9 (27.15 MPa), Z-CCK (27.06 MPa), Z-IeC (21.88 MPa), Z-VDα (21.53 MPa), Z-VM7 (20.31 MPa), and Z-Ids (3.28 MPa), which was indicative of relative survival probability of bond strength in Figure 2 (B) and  Table 2. The specimen demonstrated adhesive type of bond failure at the interface upon visually observed stereo-micrograph. The SEM photomicrograph of the fracture specimen revealed predominantly adhesive failure at the interfacial adherence zone with minute amount of ceramic particles on the irregularity surface of zirconia (Fig. 3A,C,E,G,I,K). The SEM photomicrograph at the interface revealed harmonized inter-digitation between zirconia and ceramic (Fig. 3B,D,F,H,J), except for the group of Z-IdS that indicated micro-gap at the interface as seen in Figure 3L.

**Table 2:**
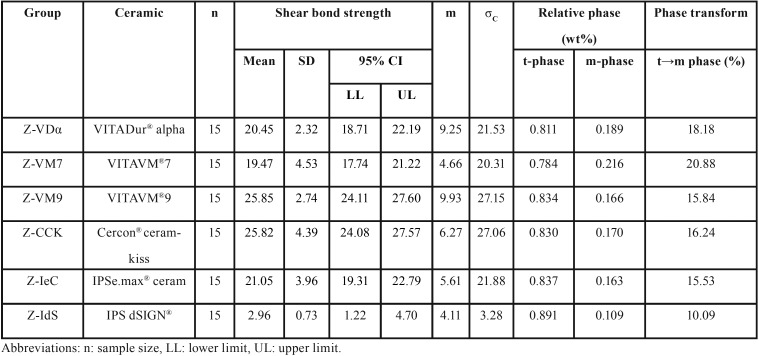
Mean, standard deviation (SD), 95% confidential interval (CI), Weibull’s modulus (m), characteristic strength (σo), relative phase content (wt%), and percentage of phase transformation (%) for shear bond strength (MPa) of zirconia veneered with VITADur® alpha (Z- VDα), VITAVM®7 (Z-VM7), IPSe.max® ceram (Z-IeC) , VITAVM®9 (Z-VM9), Cercon® ceramkiss (Z-CCK), and IPS dSIGN® (Z-IdS).

**Table 3:**
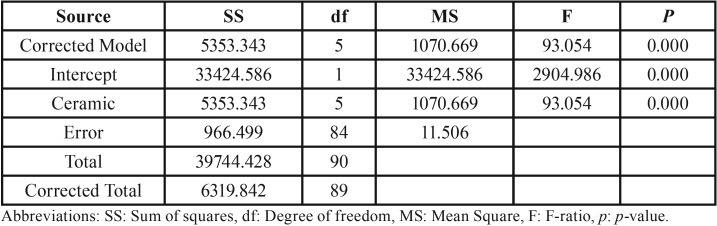
An analysis of variance (ANOVA) of shear bond strength for ceramic veneered zirconia.

**Table 4:**
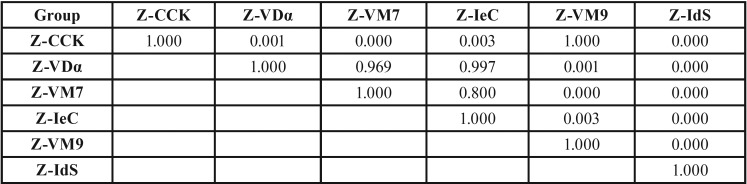
Tukey HSD multiple comparisons of shear bond strength among the groups of zirconia veneered with VITADur® alpha (Z- VDα), VITAVM®7 (Z-VM7), IPSe.max® ceram (Z-IeC), VITAVM®9 (Z-VM9), Cercon® ceramkiss (Z-CCK), and IPS dSIGN® (Z-IdS).

**Figure 4:**
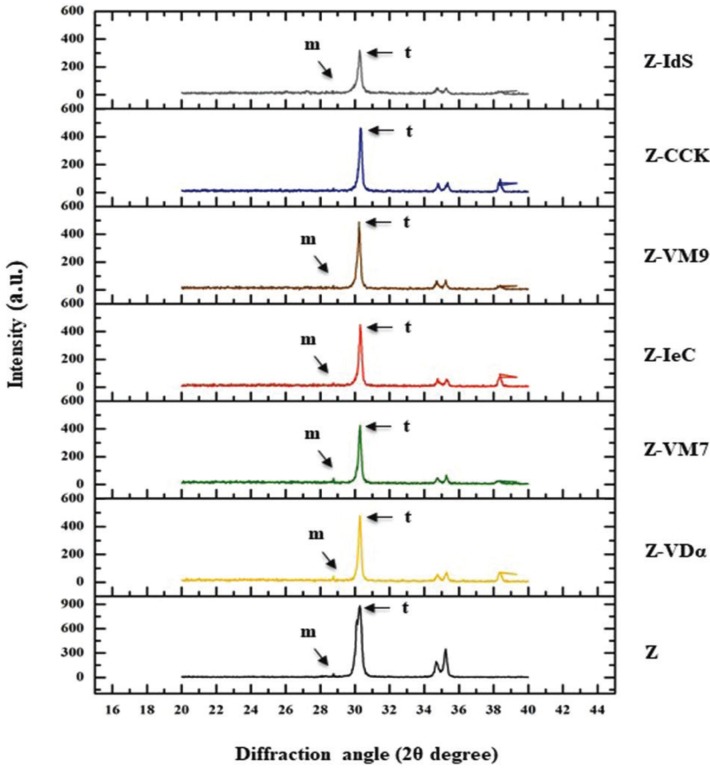
X-Ray diffraction analysis pattern of zirconia (Z) and zirconia veneered with VITADur® alpha (Z- VDα), VITAVM®7 (Z-VM7), IPSe.max® ceram (Z-IeC), VITAVM®9 (Z-VM9), Cercon® ceramkiss (Z-CCK), and IPS dSIGN® (Z-IdS).

The x-ray diffraction patterns revealed that most of crystal structure of tetragonal (t) phase with minor amount of monoclinic (m) phase in every group as shown in Figure 4. The major peaks of tetragonal phase were observed at the diffraction angle (2θ degree) of 30.29° and 34.75°. The dominant peak intensity at 30.29° corresponded to the (111) crystallographic plane of the tetragonal phase, as indicated from the X-ray diffraction standard file of zirconium oxide. The minor monoclinic phases were detected at the diffraction angle of 28.75° and 34.75°, which corresponded to the monoclinic (111) and monoclini (11ī) crystallographic plane as anticipated by the X-ray diffraction standard file. The relative concentration (wt.%) of monoclinic phase regarding the total zirconia phase revealed the variation in the amount of the phase transformation from t-phase to m-phase because of different types of veneering ceramic bonded to zirconia ( Table 2). The t- to m-phase transformation were 15.84% for Z-VM9, 16.24% for Z-CCK, 15.53% for Z-IeC, 18.16% for Z-VDα, 20.88% for Z-VM7, and 10.09% for Z-Ids group. The amount of t→m phase transformation was relatively associated with the CTE difference between veneering ceramic and zirconia.

## Discussion

This study indicated that bond strength of ceramic veneered zirconia is affected by veneering ceramics that possess different CTE values. Thus, null hypothesis was rejected. The CTE displays a major role in bond strength of ceramic veneered zirconia (17,18). The CTE difference principally influences the interfacial stress that causes crazing and ceramic delamination. A compatible CTE of veneering ceramic to zirconia results in an appropriated bond strength. All tested groups in this study exhibited the mean bond strength in the range of 19.47-25.85 MPa, except for Z-IdS, which is consistent with other studies (14,15,28). The significant highest bond strengths for both Z-CCK and Z-VM9 indicated that a compatible CTE promotes favorable bond strength. The CTE difference of 0.77×10-6/ oC for CCK and 0.87×10-6/ oC for VM9 were lower than the CTE of zirconia, which resulted in a development of desirable residual compressive stress to confer the bond strength (18,24). Bond strengths of Z-VM7 and Z-VDα were significantly lower than that of Z-CCK and Z-VM9 groups. This indicated that the CTE difference values for both groups were extremely large and led to the compromised shear bond strength. The CTE of difference of 2.93×10-6/ oC for VM7 and 2.97×10-6/ oC for VDα was lower than the CTE of zirconia, which resulted in undesirable accumulation of residual stress that induced tangential cracks upon ceramic cooling, leading to tensile stresses directly outward and perpendicular to the veneering ceramic along with resulting in a lowering of the shear bond strength as supported by other studies (10,11,26). In fact, both VM7 and VDα ceramics were manufactured for using with alumina ceramic substructures that possess a CTE in the range of 7.2-7.9 ×10-6/ oC. This study indicated the possibility of using either VM7 or VDα to veneer on zirconia, but the bond strength was not achieved entirely. The CTE of the IeC was 0.94×10-6/ oC lower than that of zirconia, which indicated a significant lower bond strength than both Z-CCK and Z-VM9, but comparable with both Z-VM7 and Z-VDα. This indicated that the CTE difference for Z-IeC was not capable of promoting favorable bond strength. This may relate to the fact that the composition of IeC is nano-fluoroapatite glass ceramic, which was deliberately produced for lithium di-silicate glass ceramic substructure. Although, the IeC was capable of veneering zirconia, but favorable bond strength was not fully accomplished. The CTE of the IdS was 2.15×10-6/ oC higher than that of zirconia, which exhibited a significantly lowest bond strength. This unfavorable bond strength was expected because of IdS possessed in CTE was much higher than the CTE of zirconia that resulted in radial stress, which was generated directly to the ceramic veneer and inhibited successful bonding (24,28). The study was supported by other studies that a CTE mismatch for approximately 2.0x10-6/οC resulted in spontaneous de-bonding after firing (25,28) with the CTE mismatch greater than 1.0 x10-6/οC having a high tendency to fail in clinical use (12).

The CTE’s difference principally influenced the interfacial residual stress that reflected on the amount of t→m phase transformation of zirconia (24). The residual stress generated from the CTE difference of Z-CCK, and Z-VM9 resulted in 16.24%, and 15.84% of phase transformation, which could have a compressive effect on the bond strength. The residual stress generated from CTE difference of Z-VDα and Z-VM7 resulted in 18.18%, and 20.88% of phase transformation, which possibly induced tangential cracks and initiated tensile stresses on veneering ceramic to engender unfavorable bond strength. The high amount of t→m phase transformation may nucleate micro-cracks in the glass phase of veneering ceramic to facilitate the ease of bond failure (5). The residual stress generated from CTE difference of Z-IeC resulted in 15.53% of phase transformation, which indicated that it was less capable of having a compressive effect to favor the bond strength, as compared to Z-CCK and Z-VM9. The amount of 10.08% phase transformation for Z-IdS indicated a minimal effect of residual stress to effectively induce phase transformation since the radial stress dominantly inhibited suitable bond.

To generate suitable levels of residual stress for promptly bond strength, appropriate CTE difference need to be primarily considered (26). This study clearly evidenced that the CTE for veneering ceramic of 0.77-0.87×10-6/ oC below the CTE of zirconia effectively conferred favorable and reliable bond strength, which was consistent with the other studies (25,26,28). Although the ideal CTE difference for ceramic veneering zirconia has not been established. The selection of ceramic for veneering zirconia must be firstly considered with its’ CTE below the CTE of zirconia as recommended in this study to achieve favorable bond strength.

## Conclusions

This investigation described the role of CTE in bond strength of ceramic veneered to Y-TZP. The study proved that bond strength of ceramic veneered zirconia was influenced by the CTE’s difference between zirconia and veneering ceramic. An extreme CTE difference between veneering ceramic and zirconia did not render favorable bond, but resulted in a higher stress inducing phase transformation of zirconia to facilitate the ease of bond failure. Proper selection of veneering ceramic based on appropriated CTE difference is extremely crucial to assure durable bond strength of ceramic fused with zirconia. The CTE of veneering ceramic less than that of zirconia for approximately 0.77-0.87X/ oC was suggested to provide suitable residual compressive stress for conferring a favorable bond strength.
